# Elements of General and Pathological Anatomy; Presenting a View of the Present State of Knowledge in These Branches of Science

**Published:** 1852-01

**Authors:** 


### Part 2—Reviews and Notices of New Works.

ARTICLE I.

*Elements of General and Pathological Anatomy ; Presenting a view of the present state of Knowledge in these Branches of Science.* By DAVID CRAIGIE, M. D., F. R. S. E., Fellow of the Royal College of Physicians, of Edinburg, &c. Second Edition, enlarged, revised, and improved , ps. 1072 octavo. Philadelphia : Lindsay & Blakiston, 1851, (from the Publishers through S. C. Griggs & Co., Chicago.)

This is a beautiful book so far as the style of its publication is concerned. We were a little surprised that so important and so elegant an Edinburg work should have been published in this country without an American editor's name being attached, as it would have given some aspiring young man a good opportunity for that kind of notoriety which many of our Eastern friends have attained ; not a few of whom may be found in the city where this book is published. But we find the edition is got up under the supervision of its own author which is certainly far better. We have very little faith in the utility or propriety of these literary God-fathers. We would much rather a work should be suffered to stand upon its own merits, and that the editors decline the distinction of authorship until they are able to produce works of their own composition. True, this might forever blot some names off of the scroll of fame, and yet we are not sure but they would be better *off*.

On looking at the last page of the work we see, by the imprint, that the printing has been done in Scotland, which shows the enterprise of the distinguished publishers, if it does not argue very strongly their support of home manufactures. The typography does no discredit to either Edinburg or the publishers.

As to the merits of the work we are unable to speak further than to say that its subjects are systematically arranged, for we have just received the copy.

We will endeavor to speak of it more fully hereafter; in the mean time we advise our readers to purchase a copy, read and judge for themselves, and we return the publishers our thanks.

